# Microbial cell-free DNA for rapid pathogen identification in clinical diagnostics: a proof of concept study

**DOI:** 10.1186/s13073-026-01700-3

**Published:** 2026-06-19

**Authors:** Micha Banz, Stefan Hagel, Sebastian Freiburger, Frederik Huhn, Merlin Krause, Stefan Glöckner, Bettina Löffler, Dennis Nurjadi, Uthayakumar Muthukumarasamy, Mateusz Jundzill, Achim J. Kaasch, Mathias W. Pletz, Christian Brandt

**Affiliations:** 1 Institute for Infectious Diseases and Infection Control, Jena University Hospital, Jena, Germany; 2 Department of Gastroenterology, Hepatology, and Infectious Diseases, Clinic of Internal Medicine IV, Jena University Hospital, Jena, Germany; 3 Department of Cardiothoracic Surgery, Friedrich-Schiller-University Jena, Jena, Germany; 4 Institute of Medical Microbiology, Jena University Hospital, Jena, Germany; 5 Institute of Medical Microbiology, University of Lübeck and University Hospital Schleswig-Holstein, Lübeck, Germany; 6 Institute of Medical Microbiology and Hospital Hygiene, Medical Facultyof the Otto Von Guericke, University Magdeburg, Magdeburg, Germany; 7 InfectoGnostics Research Campus, Jena, Germany

**Keywords:** Microbial cell-free DNA, Infectious disease diagnostics, Culture independent diagnostics, Multiplex sequencing, Oxford Nanopore sequencing

## Abstract

Microbial cell-free DNA (mcfDNA) enables rapid and sensitive pathogen detection via direct plasma-sequencing. Here, we present a fast, cost-effective mcfDNA multiplex workflow to complement or improve the sensitivity of microbiological diagnostics for systemic and focal infections which can be challenged by prior antibiotic treatment or non-culturable pathogens. In an explorative study of 18 patients, mcfDNA sequencing matched the clinical diagnosis in 16 cases (89%), was detected up to 16 days after starting targeted antibiotic therapy and identified *Staphylococcus aureus* in two cases of culture-negative endocarditis. By providing an open-access protocol, we aim to promote broader accessibility and globally available application.

**Supplementary Information:**

The online version contains supplementary material available at 10.1186/s13073-026-01700-3.

## Background

Severe bloodstream infections continue to pose a major diagnostic challenge due to the inherent limitations of culture-based methods, which offer delayed results and limited sensitivity, especially once antimicrobial treatment has commenced [1]. While targeted molecular assays, e.g. multiplex polymerase chain reaction (PCR), offer faster turnaround times, these methods are more expensive than blood culture, include more hands-on time and offer uncertain clinical benefits [2, 3]. These assays have also been considered hypothesis-driven and can only detect a limited panel of predefined pathogens, failing to identify rare or unexpected infectious agents. This lack of timely and specific diagnostic information often forces clinicians to rely on broad-spectrum empirical antibiotics, delaying targeted antibiotic therapy, and contributing to the rise of antimicrobial resistance [4, 5].

As an alternative, a hypothesis-free approach using metagenomic sequencing of microbial cell-free DNA (mcfDNA) has emerged as a promising tool in clinical research [6]. McfDNA consists of fragmented bacterial, viral, fungal, and parasitic DNA (approximately 50 to 350 bp) circulating in plasma. It is released during active infection, or following membrane disturbance caused by either the host immune response or the use of antimicrobial agents. Lacking the nucleosomal protection of host-derived counterparts, mcfDNA has an estimated plasma half-life of 10–15 min [7, 8]. Although this rapid clearance poses pre-analytical challenges, it also makes mcfDNA a sensitive biomarker for monitoring infection dynamics [9].

While this hypothesis-free approach provides a comprehensive view of the microbial landscape in the blood, its clinical utility critically depends on expert interpretation to distinguish the causative pathogen from commensals or contaminants [10, 11]. The ability to detect mcfDNA even after antibiotic administration offers a significant advantage for rapid, culture-independent diagnostics. Furthermore, its short half-life suggests usage as a dynamic biomarker to monitor infection activity and therapeutic response, as has been shown in infective endocarditis [12].

Currently, widespread adoption is hindered by reliance on costly and methodologically opaque external commercial platforms. These often involve logistical delays due to centralized testing, which can extend the median turnaround time from sample collection to report to approximately 2–3 days [13, 14]. Furthermore, these platforms do not report complex data to the local clinical teams, which hinders evaluation of the novel method [15]. To address these barriers, we developed and validated an open, on-site sequencing workflow that provides a cost-effective approach for direct mcfDNA detection from plasma, delivering results within approximately 12 h.

## Methods

### Aim, design of the study and patient cohort

This study aimed to develop and evaluate an accessible culture-independent sequencing workflow for the identification of mcfDNA in patients’ blood plasma using the Oxford Nanopore Technologies (ONT) sequencing platform. The protocol was optimized for clinical turnaround time and cost efficiency by sequencing multiple patient samples in a single multiplex run. Conducted as a prospective, observational feasibility study, this research took place in a routine clinical setting at Jena University Hospital, Germany. Written informed consent was obtained from all participants under a protocol approved by the local institutional review board (2024–3582-BO-A).

Between 1 st December 2024 and 1 st May 2025, we enrolled 18 adult patients with confirmed bloodstream infections or, in two cases (patients JE15 and JE30), imaging-based suspicion of blood culture-negative endocarditis. Patients with a new, microscopy-confirmed positive blood culture were identified on weekdays by the clinical microbiology laboratory. The study team then approached these patients to obtain their consent. Enrollment was subject to patient availability, willingness to participate and the ability to provide informed consent. Patient identifiers were assigned based on the order of enrollment in the primary clinical study and are non-consecutive in this subset, as only patients meeting the specific criteria for this study were included.

In all 18 patients, blood sampling for mcfDNA was performed by venipuncture at various time points following the start of targeted antibiotic treatment (ΔDays Abx → Seq). Technical replicates were processed whenever feasible (denoted as x.1 and x.2), and longitudinal follow-up samples were labeled alphabetically (a, b, c, etc.) for each consecutive sampling event. These follow-up samples were collected at approximately 48 h intervals until source control was achieved or the patient was either discharged with oral antibiotics or transferred to a secondary hospital. At each time point, two 10 mL Streck Cell-Free DNA BCT® tubes (Streck, USA) and one pair of blood culture bottles (BD BACTEC™ Lytic/10 Anaerobic/F and BD BACTEC™ Standard/10 Aerobic/F) were collected following standard protocols.

In total, 46 plasma samples were collected from these 18 patients and analyzed via mcfDNA sequencing. The study comprised two phases: First, an initial diagnostic assessment was performed in all 18 patients using samples collected within the first seven days after the initiation of targeted antibiotic therapy (t₀). Second, in two patients with deep-seated infections, sampling continued beyond day seven to enable longitudinal monitoring of the mcfDNA signal.

A detailed overview of all longitudinal sampling time points is provided in Additional file 1: Table S1.

### Sample collection and processing

Patient plasma was obtained from 10 mL of whole blood by centrifugation at 1,900 × g for 10 min at 4 °C to remove cellular components. A second centrifugation step at 16,000 × g for 10 min at 4 °C was performed to clear residual debris and longer DNA fragments. Plasma was kept at 4 °C and either immediately processed for mcfDNA isolation and sequencing or stored short-term (≤ 24 h) before downstream processing.

For mcfDNA isolation, we evaluated six commercial kits (Mag-Bind® cfDNA Kit, cobas® cfDNA Sample Preparation Kit, NucleoMag cfDNA Kit, MagMAX™ Cell-Free Total Nucleic Acid Isolation Kit, cfKapture™ Kit, and QIAamp MinElute ccfDNA Midi Kit). The QIAamp MinElute ccfDNA Midi Kit (Qiagen, Germany) was selected for its documented high recovery efficiency of short DNA fragments, scalability for processing 4–5 mL of plasma, and compatibility with low-input samples, which are critical parameters for mcfDNA analysis [15]. For each sample, DNA was isolated from 4–5 mL of plasma following the manufacturer’s protocol, which yielded mcfDNA concentrations of approx. 5 ng/mL. Eluted DNA was quantified using the Qubit™ dsDNA High Sensitivity Assay (Thermo Fisher Scientific). No host DNA depletion or size-selection steps were applied to the workflow.

For each sequencing run, a no-template control (NTC) consisting of nuclease-free water was included to monitor for potential cross-contamination and ensure workflow reliability. In addition, whole blood from healthy volunteers without reported infections in the preceding six weeks was collected using the same sampling strategy. Plasma from these donors was used for workflow development and spiking experiments and was processed identically to patient plasma samples.

### Library preparation and nanopore sequencing

Sequencing libraries were prepared using the Native Barcoding Kit 24 V14 (SQK-NBD114.24), following the official Oxford Nanopore Technologies (ONT) protocol *Human cfDNA multiplex sequencing from blood using SQK-NBD114.24*. While originally designed for PromethION Flow Cells, this protocol was adapted for the GridION platform, using FLO-MIN114 (R10.4.1) Flow Cells, to enhance performance with low input mcfDNA samples and lower operational costs. The modified protocol, including the succeeding steps are published online [16]. The primary adaptation involved adjusting the total volumes for Flow Cell priming and library loading to meet the specifications for FLO-MIN114 (R10.4.1) Flow Cells, as outlined in protocols such as the *Ligation Sequencing DNA V14 (SQK-LSK114)*.

**Fig. 1:**
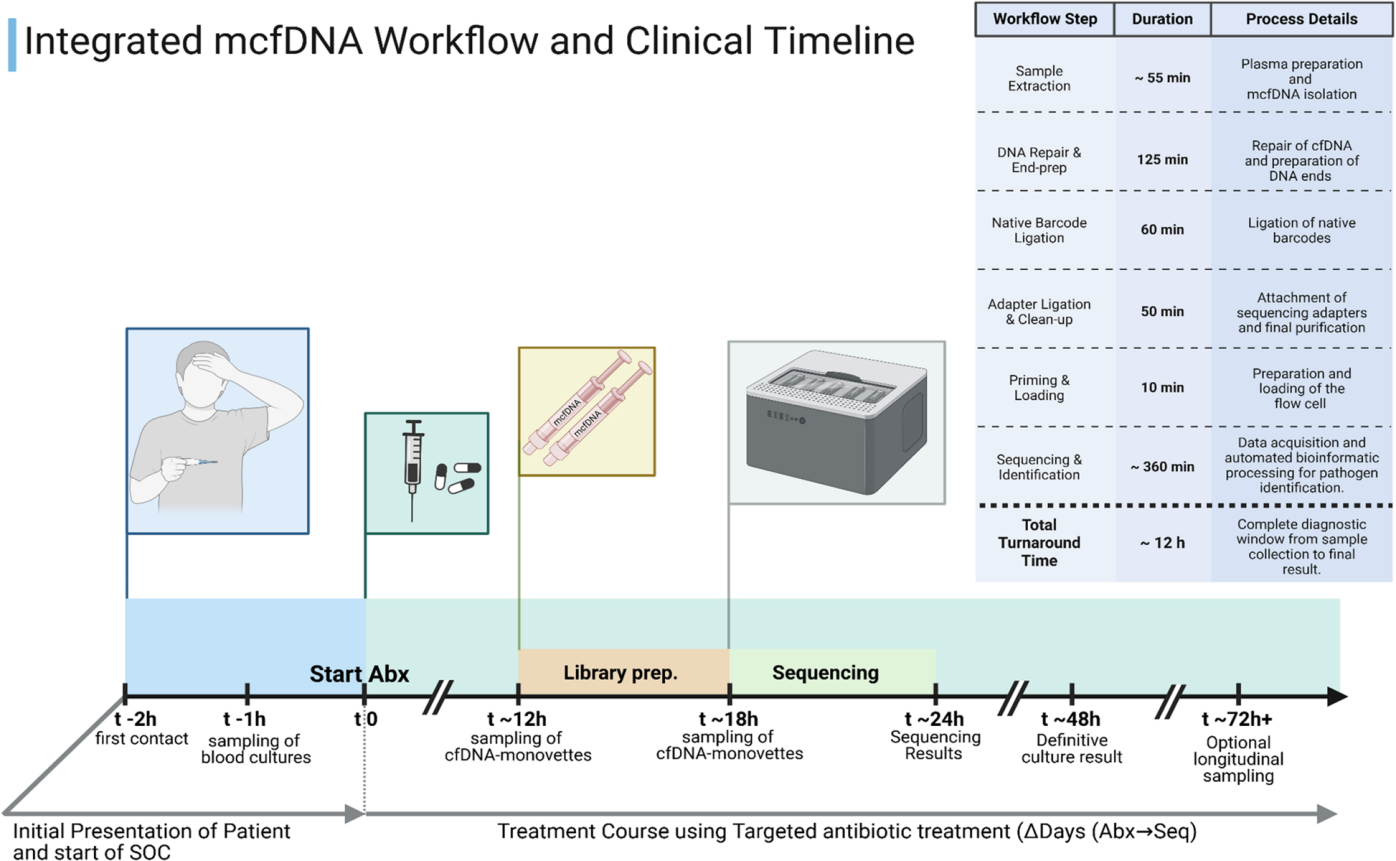
Integrated mcfDNA diagnostic workflow and clinical timeline. The schematic links the mcfDNA workflow to the clinical course. The timeline (bottom) spans from initial presentation and start of standard of care (SOC) at t − 2 h to blood culture collection at t − 1 h and initiation of targeted antibiotic treatment (Start Abx) at t0. The x-axis shows the interval between antibiotic initiation and blood sampling (ΔDays Abx → Seq). The table (top right) summarizes the technical pipeline with an overall turnaround time of ~ 12 h: extraction (~ 55 min), library preparation (245 min), and sequencing/data analysis for pathogen identification

A key finding from our optimization phase was that the total loading amount for ultra-short mcfDNA fragments on R10.4.1 Flow Cells could be increased to as high as 2,500 fmol. Following individual barcoding, libraries were quantified fluorometrically and pooled and up to six patient-samples loaded on one Flow Cell, depending on the individual DNA mass that was extracted by the isolation-protocol. Notably, normalization to achieve equimolar concentrations prior to pooling was unnecessary, as the workflow demonstrated robust sequencing performance across samples with variable mcfDNA input. The total turnaround time from sample receipt to result is approximately 12 h, comprising optimized extraction (55 min), library preparation (245 min), and a 6 h sequencing run including an automated bioinformatic analysis (Fig. 1, Table). Sequencing was performed on a GridION. MinKNOW handled real-time basecalling and demultiplexing using Dorado (v0.9.0) with the Super Accurate (SUP) model to generate FASTQ files, which were subsequently processed in our in-house workflow. Each run included a water control processed alongside clinical samples, and all work was carried out in a dedicated clean environment to minimize contamination and barcode crosstalk. Human host reads were removed prior to public release using the host-depletion workflow developed by Lataretu et al. [17] against the GRCh38/hg38 reference genome [18]. The resulting host-depleted sequencing data, Kraken2 report files (kreport), and pathogen read mappings to reference genomes are publicly available via the Open Science Framework (OSF) [19].

### Analytical workflow development and quality control

To ensure the accuracy and diagnostic reliability of sequencing results, comprehensive quality control measures were implemented throughout the workflow. Analytical sensitivity was assessed by spiking plasma from healthy volunteers with enzymatically fragmented DNA from six bacterial strains. Five of these were clinical isolates obtained from the Jena University Hospital microbiology laboratory, identified by mass spectrometry as *Enterococcus faecium*, *Escherichia coli*, *Pseudomonas aeruginosa*, *Staphylococcus aureus*, and *Acinetobacter baumannii*. The sixth strain was *Geobacillus stearothermophilus* (ATCC 12980), as a thermophilic bacterium not associated with human infection, it served as a definitive control for monitoring procedural contamination throughout the workflow. DNA was isolated using the ZymoBIOMICS™ DNA Microprep Kit (Zymo Research, USA) and enzymatically fragmented to approximately 300 bp using the NEBNext® UltraShear™ system (New England Biolabs, USA). DNA concentrations were quantified using the Qubit™ dsDNA High Sensitivity Assay (Thermo Fisher Scientific, USA) and fragment size distribution was verified with an Agilent TapeStation using the D1000 High Sensitivity ScreenTape Assay (for details refer to the online protocol and Additional file 1: Methods and Fig. S1-S3).

Serial dilutions of this fragmented bacterial DNA was spiked to blood plasma for final concentrations of 1.0, 0.5, 0.25, 0.125, 0.06, and 0.03 ng/mL and processed through the entire workflow: isolation, library preparation, sequencing, and taxonomic classification. For each concentration level, two independent replicates were prepared. In this validation setting, microbial DNA was considered detected if at least two species-specific reads passed quality filters and were correctly classified by the Kraken2 workflow [20] based on the original Kraken script [21]. In the clinical workflow, candidate pathogens were verified by mapping-based confirmation (see Section Bioinformatic analysis and contamination controls) and a comparative assessment against the run-specific baseline of NTCs and healthy volunteer samples to maintain diagnostic sensitivity and avoid false negatives from rigid taxon exclusion.

### Bioinformatic analysis and contamination controls

Starting from the demultiplexed FASTQ files generated during sequencing (as described in Section Library preparation and nanopore sequencing), a multi-step bioinformatic pipeline was established for high-confidence pathogen identification and stringent artifact removal. First, to address misclassifications arising from residual barcode sequences, reads underwent a secondary, stringent trimming step using Porechop (v0.2.4) [22]. Following this, taxonomic classification was performed using Kraken2 and species-level abundance was estimated using Bracken (v2.7) to generate a profile of candidate pathogens. To ensure high-confidence pathogen identification, we implemented a critical mapping-based validation step. Following taxonomic classification, all reads assigned by Kraken2 to a candidate pathogen and its higher taxonomic ranks were extracted using KrakenTools and subsequently mapped to its respective NCBI reference genome with Minimap2 [23]. This inclusion of parent-level reads ensures maximum sensitivity by capturing fragments that lack species-specific k-mers but align correctly to the reference sequence.

This two-stage approach was chosen to balance computational efficiency with diagnostic stringency: while primary classification allows for rapid screening, the secondary mapping serves as a formal verification against taxonomic misclassifications, localized artifacts or PCR chimeras. A pathogen was only formally confirmed if its reads showed a broad and even distribution across the reference genome, defined as reads mapping to distinct, non-overlapping genomic loci.

To further distinguish true biological signal from stochastic background noise, we established a formalized quantitative decision framework based on a Z-Score calculation (detailed in Supplementary Methods and Additional file 1: Fig. S4) [24]. Based on the mapping-confirmed background noise observed across all negative controls (mean = 0.33, standard deviation (SD) = 0.71), we defined a statistically rigorous threshold of Z ≥ 4 (*p* < 0.01) for de novo pathogen identification. This corresponds to a minimum of ≥ 4 mapping-confirmed reads, ensuring that any identified pathogen represents a significant outlier relative to the clinical background noise.

For longitudinal tracking of previously confirmed pathogens, a threshold of ≥ 2 mapping-confirmed reads was applied. This adjustment accounts for the significantly increased prior probability of pathogen presence during follow-up. To ensure specificity at these lower limits, reads were strictly required to align to distinct genomic loci, and the concurrent run-specific negative control was required to demonstrate zero reads for the target pathogen.

## Results

### Implementation of the mcfDNA sequencing protocol: cost efficiency, analytical sensitivity and quality control

We developed and validated an accessible mcfDNA sequencing workflow for the ONT GridION/MinION platform using R10.4.1 Flow Cells. The protocol is optimized for multiplexing up to six samples per Flow Cell and achieves a reagent cost of roughly €100 per patient sample. The complete, validated protocol is available online [16].

**Fig. 2:**
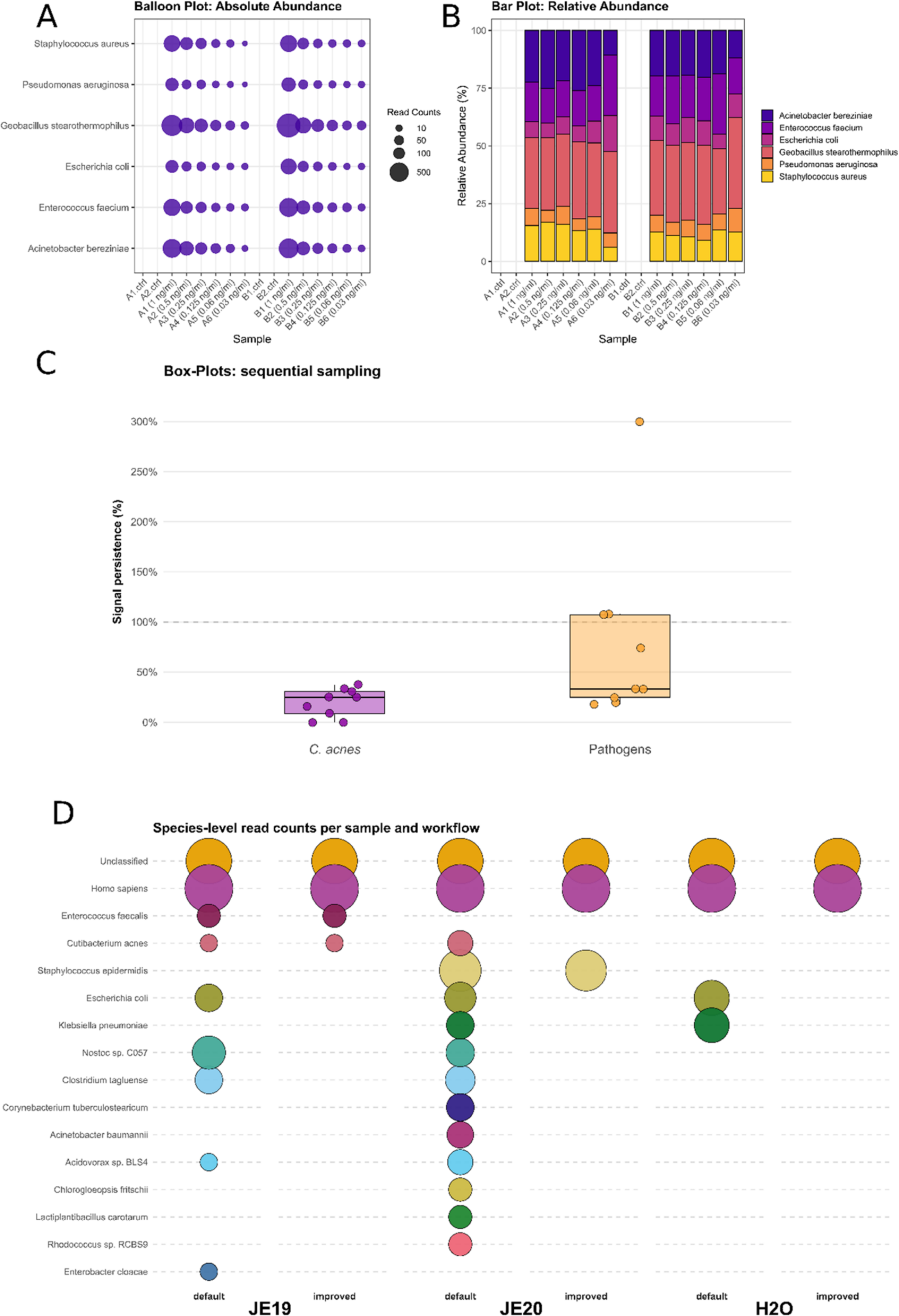
Workflow validation, from analytical sensitivity to bioinformatic optimization.** A**, **B** Analytical sensitivity and quantification in spiked plasma. Absolute read counts for six bacterial species across a serial dilution (1.00 to 0.03 ng/mL) are shown in (**A**), with corresponding relative abundances in (**B**). Both biological replicates demonstrate stable quantification over the higher concentration range and consistent detection of all species down to 0.03 ng/mL. **C** Pre-analytical contamination control with a two-tube collection strategy. Comparative analysis of microbial signal persistence between two sequential blood draws (Tube 1 vs. Tube 2). Signals assigned to the skin commensal C. acnes show a significant depletion in the second tube (paired Wilcoxon signed-rank test, *p* < 0.0045). In contrast, confirmed pathogen signals (e.g., S. aureus, K. pneumoniae) exhibit stable persistence across both tubes (paired Wilcoxon signed-rank test, *p* < 0.094), facilitating the distinction between collection-related contamination and a true bloodstream signal. **D** Impact of stringent barcode trimming on taxonomic accuracy. Species-level balloon plots compare the default and improved workflow across three runs: patient samples JE19 and JE20, and a water control (NTC). Only taxa with reads in either workflow are shown. Bubble sizes encode absolute read counts on a global scale (capped for readability)

To assess analytical sensitivity and stability, we spiked enzymatically fragmented DNA of ~ 350 bp from six bacteria -clinical *A. baumannii*, *E. faecium*, *E. coli*, *P. aeruginosa*, *S. aureus*, and non-pathogenic *G. stearothermophilus*- into plasma from two healthy volunteers. The mix contained equal mass concentrations and was diluted from 1.00 to 0.03 ng/mL. All six species were consistently detected down to 0.03 ng/mL in both replicates, and input mass correlated with recovered read counts (Fig. 2A and 2B). Relative abundances were stable at higher concentrations and became less consistent at 0.03 ng/mL, which we consider the practical boundary for quantification. No barcode cross-contamination was detected in the negative controls A1.ctrl, A2.ctrl, B1.ctrl and B2.ctrl, a known risk with small, short DNA inputs. Although we did not reach a strict technical detection limit, 0.03 ng/mL provided a pragmatic threshold for clean and reproducible detection in multiplexed samples.

A central challenge in plasma mcfDNA is the dominance of human DNA at 90–99% of reads, which leaves few microbial reads and complicates discrimination of true pathogens from environmental- or skin contaminants. Early runs detected the skin commensal *Cutibacterium acnes*, leading us to introduce a paired sampling strategy during the study. Across pairs, *C. acnes* showed a significant depletion in the second tube (paired Wilcoxon signed-rank test, *p* = 0.0045). In contrast, confirmed pathogen reads demonstrated stable persistence across both tubes (*p* = 0.094), which helped separate collection-related contamination from a true bloodstream signal (Fig. 2C).

For bioinformatic analysis, we compared two workflows. The default workflow used MinKNOW with Dorado demultiplexing and the original Kraken2 script. The improved workflow added Porechop for barcode trimming before downstream classification. The improved workflow reduced false-positive assignments by about 17% and removed background taxa that are highly likely caused by barcode misclassification and residual barcode sequence. This included spurious low-count calls such as *Nostoc* and *Rhodococcus* as well as occasional misassigned reads to common pathogens like *E. coli*, *K. pneumoniae* and *E. cloacae* (Fig. 2D). Notably, while unfiltered datasets initially showed misassigned reads at levels comparable to primary pathogens due to barcode crosstalk, the implementation of the refined bioinformatic pipeline successfully eliminated these artifacts. Following this filtering, no clinically relevant human pathogens were detected in NTCs and healthy controls, with the exception of common skin commensals (e.g., *C. acnes*). To maintain diagnostic integrity, we established a strict interpretation rule: any clinical sample must be rejected if its concurrent NTC demonstrates microbial signals other than common skin-related contaminants. Detailed read counts for all clinical samples and controls are provided in Additional file 1: Table S2.

During analytical validation we observed one consistent taxonomic discrepancy. A strain identified as *A. baumannii* by VITEK-MS was classified as *A. bereziniae* by our metagenomic next-generation sequencing (mNGS) workflow across all spiked replicates. This finding most likely reflects limitations of the VITEK-MS reference database, in which *A. bereziniae* is not represented.

### Pathogen detection and longitudinal monitoring in clinical samples

**Fig. 3:**
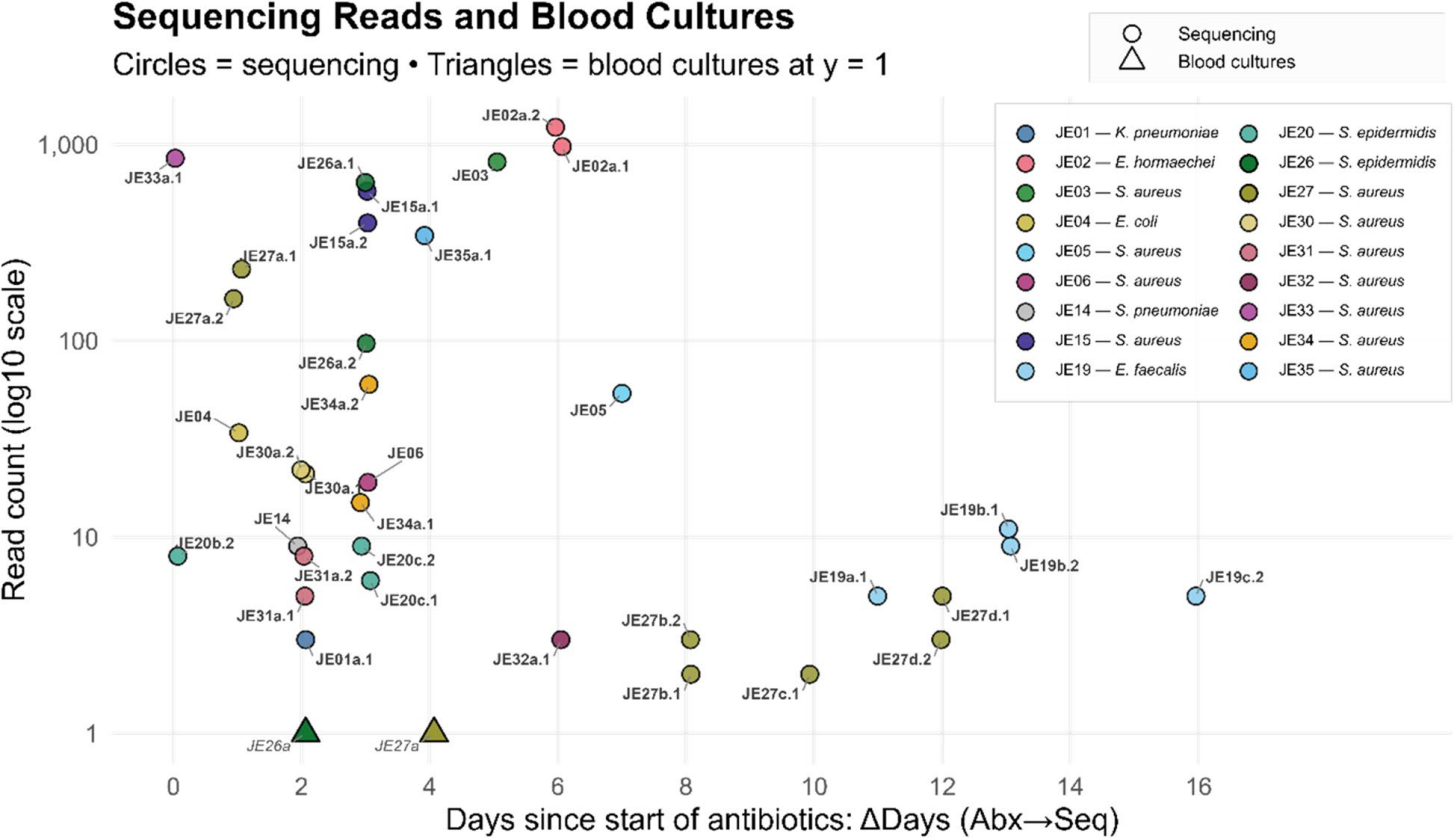
Sequencing results from patient plasma samples after initiating antibiotic therapy. Sequencing results from patient plasma samples after initiating antibiotic therapy. Pathogen-specific mcfDNA read counts per plasma sample (circles; y-axis, log10 scale of mapping-confirmed reads) and corresponding blood culture results (triangles; binary indicator plotted at y = 1 for a positive culture) are shown relative to the start of targeted antibiotic therapy (day 0). The x-axis corresponds to ΔDays (Abx → Seq) in Additional file 1: Table S1. Each circle represents a unique plasma sample. Patient IDs are labeled directly in the plot to facilitate cross-referencing with clinical metadata; alphabetical suffixes (a, b, c) denote longitudinal follow-up events, while numerical suffixes (.1,.2) indicate technical replicates

Following analytical validation, we applied the mcfDNA sequencing workflow to the clinical cohort as described in the Methods section. An overview of all sampling time points and individual sequencing results is shown in Fig. 3.

For the initial diagnostic assessment across all 18 patients, results based on confirmed reads showed high concordance with conventional microbiology. At the species level, they matched in 16 of 18 cases (89%). The overall diagnostic yield of mcfDNA sequencing was equal to that of conventional blood culture (16/18, 89%). Blood cultures yielded a pathogen in two cases where mcfDNA read counts fell below the detection threshold, whereas mcfDNA sequencing identified the causative pathogen in two cases of culture-negative endocarditis.

The two cases where mcfDNA remained below the diagnostic threshold highlight the limitations of low-abundance signals. In the first case (patient JE01), mapping-based validation yielded *Klebsiella oxytoca* as the primary candidate with 3 confirmed reads. However, this finding was viewed critically due to the concurrent detection of 2 reads assigned to *K. pneumoniae*, and ultimately, both signals fell below our Z ≥ 4 stringency requirement. Routine blood culture later isolated *K. oxytoca*. In the second case (patient JE32), 3 reads could be mapped to the reference genome, therefore falling below the threshold.

Conversely, beyond simple concordance, our workflow provided superior taxonomic resolution in patient JE02 by identifying *Enterobacter hormaechei*, whereas routine diagnostics only resolved the pathogen to the *Enterobacter cloacae complex*, a well-documented limitation of conventional mass spectrometry [25].

Of the blood cultures drawn concurrently with each mcfDNA sample, only two yielded positive results (patient JE26 at day 2 and patient JE27 at day 4). In both instances, the isolated pathogen perfectly matched the species already identified by our mcfDNA workflow.

For the two patients undergoing extended monitoring, the causative pathogen's mcfDNA remained detectable for up to 16 days. The read counts observed from day 8 onwards were significantly lower than in the initial diagnostic period, approaching background levels. Notably, the signal only became undetectable following the surgical removal of the infectious focus.

As we did not apply artificial host depletion, adaptive sequencing, or enzymatic enrichment of prokaryotic DNA, sequencing libraries contained a high proportion of human cfDNA, averaging 90–99%. Despite this immense host DNA burden, the workflow yielded sufficient pathogen reads for detection and identification.

**Fig. 4:**
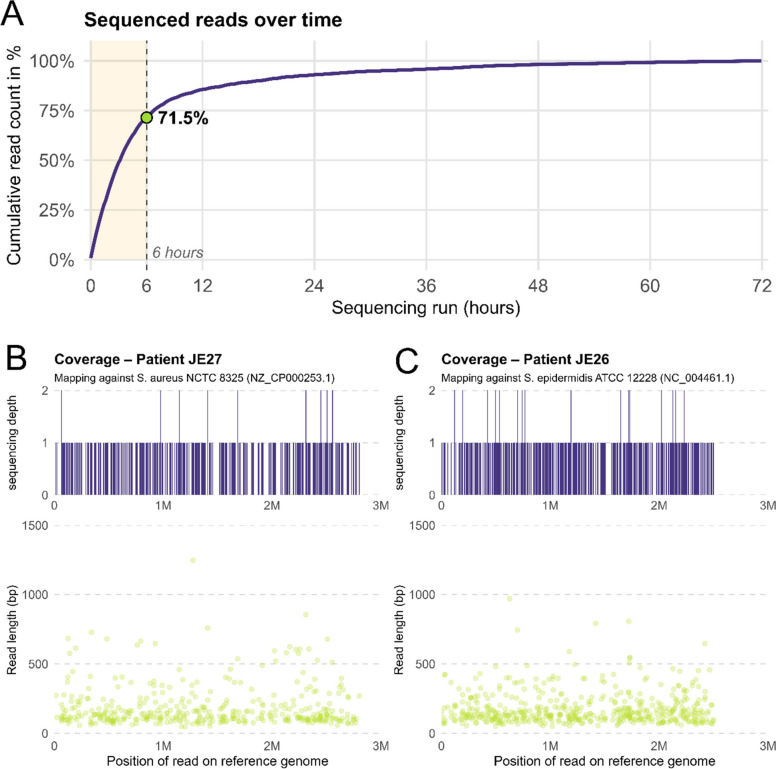
Sequencing kinetics and genome coverage analysis to confirm pathogenic hits. **A** Cumulative fraction of pathogen-specific reads over sequencing time, demonstrating a rapid increase during the first six hours of sequencing, followed by a transition to a slower accumulation phase. After 6 h, approximately 72% of the total reads observed by 72 h had already been generated. This pattern highlights the temporal dynamics of nanopore sequencing for capturing mcfDNA. **B**, **C** Confirmed reads from patient samples JE27 (Sample JE27a.1) and JE26 (Sample JE26a.1) mapped against the respective NCBI reference genomes for S. aureus NCTC 8325 (NZ_CP000253.1) and Staphylococcus epidermidis (NC_004461.1), respectively. Both mapping plots reveal uniform distribution of sequencing reads across the respective bacterial genomes, supporting unbiased pathogen detection and robust confirmation of microbial presence. The upper tracks show the mapping density (depth profile calculated via Samtools depth after alignment to the reference) [29], while the lower tracks visualize the position and length of individual mapped reads

Time-stamped analysis of the sequencing output revealed a rapid accumulation of reads during the initial six hours of the run, followed by a distinct transition to a slower, linear phase (Fig. 4:A). This slowdown directly corresponds to a decrease in the number of active pores over time, an effect likely accelerated by the short fragment size of mcfDNA. Critically, this demonstrates that sufficient data for pathogen identification is captured during this initial high-throughput phase.

Across all patient samples, the average bacterial read length was 190 bp (SD ± 150 bp), with a notable subset of reads extending up to 1,248 bp (Fig. 4:B, C). These longer fragments were confirmed to originate from the identified pathogens via BLAST analysis [26]. The even distribution of reads across the pathogen genomes, with no apparent bias or amplification hotspots, reinforces the specificity of our pipeline and makes contamination from sources like carryover PCR products highly unlikely [27, 28].

For patient JE20, who was transferred with suspected spondylodiscitis and infective endocarditis, initial piperacillin/tazobactam treatment was discontinued upon admission. In the subsequent antibiotic-free period, our mcfDNA sequencing identified *Staphylococcus epidermidis* with a strong signal (158 and 189 confirmed reads in the two technical replicates, respectively). This finding was consistent with blood cultures taken both before and after transfer, which isolated a piperacillin/tazobactam-resistant *S. epidermidis* strain. Based on the high read count and concordant culture results, *S. epidermidis* was considered the causative agent. The pathogen's mcfDNA signal remained detectable for three days after targeted antibiotic therapy with ceftriaxone and daptomycin was initiated (t_0_).

## Discussion

Our study introduces a widely accessible Nanopore workflow that complements conventional diagnostics. This approach addresses reduced culture sensitivity, particularly following antibiotic use, while leveraging metagenomics to detect non-culturable pathogens.

While early work in high-acuity settings like the intensive-care unit (ICU) established the clinical feasibility of mNGS in sepsis patients [9, 30], widespread adoption has been hampered by the excessive cost and logistical delays of centralized send-out testing. Our on-site model overcomes these barriers for infectious diseases with systemic or local focus, delivering results in approximately 12 h for as low as 100€ per sample. This offers a significantly faster and more cost-effective alternative to currently available commercial tests and is even less expensive than several commercially available multiplex PCR-solutions. This approach empowers the local clinical team to directly integrate complex metagenomic data with the patient’s immediate context, fostering more timely and informed clinical decision-making.

A strength of our workflow is its potential for enhanced taxonomic precision compared to conventional diagnostics. This was demonstrated by the reclassification of a clinical isolate initially identified as *A. baumannii* to *A. bereziniae*. This finding highlights a well-documented challenge for routine methods, which can struggle to accurately differentiate between closely related *Acinetobacter* species, including those within the clinically important *Acinetobacter calcoaceticus-baumannii* (Acb) complex [31].

Similarly, our workflow provided higher resolution by identifying *E. hormaechei* at the species level, whereas standard diagnostics had only resolved the pathogen to the broader *E. cloacae complex*. This granular identification is particularly relevant as commercial mass spectrometry systems often struggle to accurately discriminate between species of the *E. cloacae complex* and frequently provide only genus or complex level assignments [25, 32].

In contrast, the case of patient JE01 illustrates that low counts are difficult to interpret and must always be evaluated within the broader context of the sequencing run. In this instance, the detection of only two mapped reads led to an assignment of *K. pneumoniae* while a subsequent directed sequencing experiment of the clinical isolate confirmed *K. oxytoca*. This discrepancy likely stems from the fact that the few available mcfDNA-fragments showed high homology to multiple species within the Klebsiella genus, which reflects a known challenge in metagenomics when distinguishing between highly homologous genomes, especially in the Klebsiella family [33, 34].

To address such limitations, our workflow utilizes the signal to noise ratio instead of rigid numerical thresholds. Confirmed pathogens in this study typically demonstrated at least a fivefold higher abundance than background taxa in the initial classification, which further increased to a tenfold difference after mapping based validation. To resolve cases where the signal strength remains below these margins, a diagnostic escalation such as re-sequencing a backup sample in singleplex mode would be the appropriate clinical step to achieve high-confidence, species-level identification.

A key advantage of the on-site model is its flexibility, allowing the sequencing strategy to be tailored to clinical urgency and integrated into routine diagnostics. While cost-effective multiplexing is suitable for routine screening, the workflow enables a rapid pivot to singleplex sequencing for high-priority cases. Dedicating an entire Flow Cell to a single patient shortens turnaround time and increases sequencing depth, at the expense of a higher per-sample cost. Although the ratio between pathogen and background DNA remains constant, higher depth increases the number of pathogen reads and thereby the statistical confidence of mapping-based validation, including the ability to demonstrate reads distributed across distinct genomic loci. Based on our dataset, a target of approximately 20–50 pathogen-specific reads was sufficient for robust species-level identification. This increased depth is particularly relevant for low-abundance signals in deep-seated infections and in patients receiving prolonged antibiotic therapy, where mcfDNA levels can be profoundly low.

Our ability to monitor mcfDNA kinetics for over 16 days post-treatment initiation highlights its potential to evolve from simple pathogen identification towards a quantitative liquid biopsy for infection that could be used to guide and personalize the individual treatment regimen.

The observed decay of the mcfDNA signal suggests its potential as a dynamic biomarker for therapeutic efficacy which is in line with current understanding of mcfDNA physiology [7]. While enzymatic spike-ins served as a technical benchmark for protocol optimization, our clinical findings of a 190 bp average read length and fragments exceeding 1200 bp demonstrate that the workflow effectively identifies natural mcfDNA across a size spectrum that shares significant overlap with our technical models. This introduces the compelling prospect of defining a state of *minimal residual disease* (MRD) for bacterial infections, a framework where quantitative monitoring could guide therapy duration, predict relapse risk, or non-invasively confirm successful source control [12]. If confirmed by randomized trials, such a personalized approach would probably decrease antibiotic consumption, length of stay, costs and antibiotic side effects without increasing relapse rates. While our current multiplexed workflow serves as a cost-effective screening tool, the interpretation of low-abundance signals during longitudinal monitoring can be further strengthened by increasing sequencing depth. For clinical applications demanding high-confidence tracking of declining mcfDNA levels, such as confirming source control or personalizing therapy duration, transitioning to singleplex sequencing or higher-capacity flow cells (e.g., PromethION) provides the crucial data density to maintain rigorous statistical confidence at the detection limit.

Our study also provides practical solutions to persistent methodological challenges. In particular, the importance of a standardized pre-analytical workflow for mcfDNA-analysis cannot be overstated. In line with existing literature, we confirmed that the use of specialized blood monovettes is crucial for preserving mcfDNA integrity compared to standard K3-EDTA tubes used for clinical chemistry (Additional file 1: Table S2), reinforcing the need for a standardized pre-analytical workflow [22, 35]. Furthermore, the two-tube collection strategy offers a simple method to minimize contamination from skin flora, while the non-human spike-in *G. stearothermophilus* serves as an invaluable internal control to ensure workflow fidelity from extraction to analysis [36].

Despite these advances, our study has limitations. The results from our small cohort require validation in larger, prospective trials to fully define clinical utility. Additionally, while currently effective, our reliance on visual inspection for alignment validation introduces a potential for operator bias, and future work will focus on developing an automated script to standardize this critical confirmation step. Furthermore, a significant current limitation of our workflow is the inability to reliably detect antimicrobial resistance (AMR) genes directly from the low abundance mcfDNA signal, which is a critical component for guiding targeted antibiotic therapy. This conclusion is consistent with other recent work, which highlights that the sparse nature of mNGS data makes reliable AMR detection a significant challenge that requires stringent filtering to ensure accuracy [37, 38]. While targeted strategies using enrichment for specific genetic markers have demonstrated the potential for AMR detection in mcfDNA [37], our framework prioritizes rapid, unbiased pathogen identification. Within this non-targeted framework, the detection of a broad range of resistance markers would currently require a sequencing depth that exceeds the time and cost constraints of a rapid diagnostic tool. Though future projects are already focusing on the inclusion of analysis of AMR genes. Consequently, our workflow serves as a complementary diagnostic tool, while conventional microbiology remains essential for susceptibility testing. Future work must therefore focus on validating the MRD concept and integrating advancements for sensitive AMR gene detection. As the field evolves, our open framework is also well-suited to incorporate alternative analytical approaches: while methods such as marker gene profiling or machine learning offer potential improvements, they often involve trade-offs in speed, complexity, and computational demand that must be carefully balanced to maintain clinical utility.

## Conclusions

This study describes and validates an optimized, on-site Nanopore sequencing workflow for the rapid, cost-efficient, and specific detection of mcfDNA from plasma. Our method demonstrated high analytical sensitivity, achieved excellent diagnostic concordance with conventional blood cultures, and proved its clinical value by identifying the causative pathogen in two culture-negative endocarditis cases. Furthermore, the workflow was effective for longitudinal monitoring in patients with deep-seated infections and could potentially be used to personalize treatment duration. By providing a fully validated and accessible protocol, this work removes significant barriers to implementation, paving the way for the broader adoption of mcfDNA sequencing in routine clinical diagnostics for infectious diseases.

## Supplementary Information

Supplementary Material 1.

## Data Availability

The host-depleted metagenomicsequencing data generated during this study are publicly available via the Open Science Framework (OSF). To protect patient privacy, all reads mapping to the human reference genome were removed prior to upload. The dataset includes demultiplexed FASTQ files and associated clinical metadata. To access these materials, please navigate to the OSF project repository using the link provided in reference [19]. From there, the data can be viewed and downloaded directly via the repository’s interface, where all files are organized into structured directories containing the sequencing output and metadata. The complete bioinformatic workflow and custom scripts used in this study are available on GitHub and permanently archived in Zenodo [39].

## Abbreviations

AMR: Antimicrobial resistance
BCT: Blood collection tube (from "streck cell-free dna bct® tubes")
BLAST: Basic local alignment search tool
bp: Base pair
cfDNA: Cell-free dna
EDTA: Ethylenediaminetetraacetic acid
fmol: Femtomole
ICU: Intensive care unit
LOD: Limit of detection
mcfDNA: Microbial cell-free dna
mL: Milliliters
MRD: Minimal residual disease
NCBI: National center for biotechnology information
mNGS: Metagenomic next-generation sequencing
ONT: Oxford nanopore technologies
PCR: Polymerase chain reaction
SD: Standard deviation
SUP: Super-accuracy (from "sup basecalling model")
TAT: Total turnaround time
