# Supplementary material for: Microbial cell-free DNA for rapid pathogen identification in clinical diagnostics: a proof of concept study
Source: Genome Med. 2026 Jun 19;18:88. doi: 10.1186/s13073-026-01700-3 (PMC13281617; doi:10.1186/s13073-026-01700-3)
Supplement: Supplementary file 1 — Supplementary Material 1. [file 13073_2026_1700_MOESM1_ESM.pdf]

Additional file 1: Supplementary Appendix

Table S1: Overview of patient characteristics and mcfDNA sequencing results.

The table details findings from 46 plasma samples from 18 patients with confirmed or suspected bloodstream or endocardial infections. "ΔDays (Abx→Seq)" indicates the number of days between the initiation of targeted antibiotic therapy and blood sampling. "Pathogen" refers to the species identified by mcfDNA sequencing, while "Confirmation" specifies the result from the corresponding clinical reference method (e.g., blood culture or valve PCR). Any discrepancies in species-level identification are noted in parentheses.

Patient ID	Sample ID	Δdays (Abx->Seq)	Reads	Pathogen	Confirmation
JE01	JE01a.1	2	2	K. pneumoniae	Blood culture (K. oxytoca)
JE01	JE01a.2	2	0	K. pneumoniae	Blood culture (K. oxytoca)
JE02	JE02a.1	6	977	E. hormaechei	Blood culture (E. cloacae complex)
JE02	JE02a.2	6	1226	E. hormaechei	Blood culture (E. cloacae complex)
JE03	JE03a.1	5	977	S. aureus	Blood culture
JE04	JE04a.1	1	34	E. coli	Blood culture
JE05	JE05a.1	7	54	S. aureus	Blood culture
JE06	JE06a.1	3	19	S. aureus	Blood culture
JE14	JE14a.1	2	9	S. pneumoniae	Blood culture
JE15	JE15a.1	3	579	S. aureus	Valve PCR
JE15	JE15a.2	3	399	S. aureus	Valve PCR
JE19	JE19a.1	11	5	E. faecalis	Blood culture
JE19	JE19a.2	11	1	E. faecalis	Blood culture
JE19	JE19b.1	13	11	E. faecalis	Blood culture

JE19	JE19b.2	13	9	E. faecalis	Blood culture
JE19	JE19c.1	16	2	E. faecalis	Blood culture
JE19	JE19c.2	16	5	E. faecalis	Blood culture
JE20	JE20a.1	NA	158	S. epidermidis	Blood culture
JE20	JE20a.2	NA	189	S. epidermidis	Blood culture
JE20	JE20b.1	0	7	S. epidermidis	Blood culture
JE20	JE20b.2	0	8	S. epidermidis	Blood culture
JE20	JE20c.1	3	6	S. epidermidis	Blood culture
JE20	JE20c.2	3	9	S. epidermidis	Blood culture
JE26	JE26a.1	3	642	S. epidermidis	Blood culture
JE26	JE26a.2	3	97	S. epidermidis	Blood culture
JE27	JE27a.1	1	232	S. aureus	Blood culture
JE27	JE27a.2	1	164	S. aureus	Blood culture
JE27	JE27b.1	8	2	S. aureus	Blood culture
JE27	JE27b.2	8	3	S. aureus	Blood culture
JE27	JE27c.1	10	2	S. aureus	Blood culture
JE27	JE27c.2	10	0	S. aureus	Blood culture
JE27	JE27d.1	12	5	S. aureus	Blood culture
JE27	JE27d.2	12	3	S. aureus	Blood culture
JE27	JE27e.1	15	0	S. aureus	Blood culture
JE27	JE27e.2	15	1	S. aureus	Blood culture
JE30	JE30a.1	2	21	S. aureus	Valve PCR
JE30	JE30a.2	2	22	S. aureus	Valve PCR
JE31	JE31a.1	2	5	S. aureus	Blood culture
JE31	JE31a.2	2	8	S. aureus	Blood culture
JE32	JE32a.1	6	3	S. aureus	Blood culture
JE32	JE32a.2	6	0	S. aureus	Blood culture
JE33	JE33a.1	0	1065	S. aureus	Blood culture
JE33	JE33a.2	0	852	S. aureus	Blood culture
JE34	JE34a.1	3	15	S. aureus	Blood culture

JE34	JE34a.2	3	60	S. aureus	Blood culture
JE35	JE35a.1	4	344	S. aureus	Blood culture

Table S2: Sequencing read summary including controls

The table reports per-sample sequencing output and classification results, including total reads, the number and percentage of reads assigned to the human host, unclassified reads, and non-human reads. “Candidate reads (Kraken2)” lists reads assigned by Kraken2 to the reported pathogen(s), while “Confirmed reads (mapping)” lists reads mapping to the corresponding pathogen reference genome(s) as part of the mapping-based validation. Sample identifiers include patient samples (JE…), nuclease-free water no-template controls (H2O.1–H2O.6), and healthy volunteer samples (HV.1–HV.3)

Sample-ID	Total reads	Human reads, n	Human reads, %	Unclassified reads, n	Unclassified reads, %	Non-human reads, n	Non-human reads, %	Candidate reads (Kraken2), n	Confirmed reads (mapping), n
H2O.1	3.805	3.420	89,88%	385	10,12%	0	0,0000%	NA	0
H2O.2	9.118	8.067	88,47%	1.050	11,52%	1	0,0110%	NA	0
H2O.3	3.367	2.998	89,04%	365	10,84%	4	0,1188%	NA	Moraxella osloensis (2)
H2O.4	108.967	95.954	88,06%	13.010	11,94%	3	0,0028%	NA	0Cutibacterium acnes (1)
H2O.5	84.418	73.050	86,53%	11.360	13,46%	8	0,0095%	NA	0
H2O.6	2.534	2.151	84,89%	381	15,04%	2	0,0789%	NA	0
HV.1	229.780	225.561	98,16%	4.219	1,84%	0	0,0000%	NA	0
HV.2	177.583	174.151	98,07%	3.428	1,93%	4	0,0023%	NA	Lactococcus garvieae (1)
HV.3	173.633	170.624	98,27%	3.008	1,73%	1	0,0006%	NA	0
JE01a.1	16.870.626	15.387.329	91,21%	1.483.062	8,79%	235	0,0014%	Klebsiella pneumoniae (3), Klebsiella oxytoca (1)	Klebsiella pneumoniae (2), Klebsiella oxytoca (3)
JE01a.2	17.063.069	15.757.002	92,35%	1.306.000	7,65%	67	0,0004%	NA	NA
JE02a.1	17.854.332	16.896.909	94,64%	955.972	5,35%	1451	0,0081%	Enterobacter hormaechei (182)	Enterobacter hormaechei (977)
JE02a.2	23.266.879	22.594.215	97,11%	670.891	2,88%	1773	0,0076%	Enterobacter hormaechei (206)	Enterobacter hormaechei (1226)
JE03	2.507.662	2.345.831	93,55%	160.345	6,39%	1486	0,0593%	Staphylococcus aureus (152)	Staphylococcus aureus (977)
JE04	1.773.974	1.721.139	97,02%	52.723	2,97%	112	0,0063%	Escherichia coli (20)	Escherichia coli (34)
JE05	4.842.295	4.706.646	97,20%	135.467	2,80%	182	0,0038%	Staphylococcus	Staphylococcus

								aureus (14)	aureus (54)
JE06	5.488.791	5.296.885	96,50%	191.852	3,50%	54	0,0010%	Staphylococcus aureus (5)	Staphylococcus aureus (19)
JE14	1.430.961	1.394.250	97,43%	36.681	2,56%	30	0,0021%	Streptococcus pneumoniae (9)	Streptococcus pneumoniae (9)
JE15a.1	2.054.366	1.957.088	95,26%	96.678	4,71%	600	0,0292%	Staphylococcus aureus (121)	Staphylococcus aureus (579)
JE15a.2	1.435.427	1.351.450	94,15%	83.555	5,82%	422	0,0294%	Staphylococcus aureus (84)	Staphylococcus aureus (399)
JE19a.1	885.421	857.081	96,80%	28.329	3,20%	11	0,0012%	Enterococcus faecalis (5)	Enterococcus faecalis (5)
JE19a.2	733.962	712.634	97,09%	21.325	2,91%	3	0,0004%	Enterococcus faecalis (1)	Enterococcus faecalis (1)
JE19b.1	1.504.446	1.461.998	97,18%	42.430	2,82%	18	0,0012%	Enterococcus faecalis (9)	Enterococcus faecalis (11)
JE19b.2	2.590.019	2.532.208	97,77%	57.798	2,23%	13	0,0005%	Enterococcus faecalis (8)	Enterococcus faecalis (9)
JE19c.1	826.894	802.407	97,04%	24.482	2,96%	5	0,0006%	NA	NA
JE19c.2	1.085.208	1.055.805	97,29%	29.387	2,71%	16	0,0015%	Enterococcus faecalis (5)	Enterococcus faecalis (5)
JE20a.1	1.245.110	1.217.001	97,74%	27.939	2,24%	170	0,0137%	Staphylococcus epidermidis (159)	Staphylococcus epidermidis (158)
JE20a.2	1.536.199	1.499.766	97,63%	36.223	2,36%	210	0,0137%	Staphylococcus epidermidis (193)	Staphylococcus epidermidis (189)
JE20b.1	420.925	410.499	97,52%	10.416	2,47%	10	0,0024%	Staphylococcus epidermidis (7)	Staphylococcus epidermidis (7)
JE20b.2	473.693	462.353	97,61%	11.330	2,39%	10	0,0021%	Staphylococcus epidermidis (8)	Staphylococcus epidermidis (8)
JE20c.1	623.715	605.612	97,10%	18.092	2,90%	11	0,0018%	Staphylococcus epidermidis (6)	Staphylococcus epidermidis (6)
JE20c.2	779.050	762.179	97,83%	16.850	2,16%	21	0,0027%	Staphylococcus epidermidis (9)	Staphylococcus epidermidis (9)
JE26a.1	456.280	443.609	97,22%	11.903	2,61%	768	0,1683%	Staphylococcus epidermidis (680)	Staphylococcus epidermidis (642)
JE26a.2	90.907	88.262	97,09%	2.528	2,78%	117	0,1287%	Staphylococcus epidermidis (111)	Staphylococcus epidermidis (97)
JE27a.1	668.850	650.582	97,27%	17.965	2,69%	303	0,0453%	Staphylococcus aureus (128)	Staphylococcus aureus (232)
JE27a.2	470.110	454.803	96,74%	15.114	3,21%	193	0,0411%	Staphylococcus aureus (88)	Staphylococcus aureus (164)

JE27b.1	743.169	727.758	97,93%	15.347	2,07%	64	0,0086%	Staphylococcus aureus (1)	Staphylococcus aureus (2)
JE27b.2	600.693	583.073	97,07%	17.597	2,93%	23	0,0038%	Staphylococcus aureus (1)	Staphylococcus aureus (3)
JE27c.1	923.428	902.534	97,74%	20.881	2,26%	13	0,0014%	Staphylococcus aureus (1)	Staphylococcus aureus (2)
JE27c.2	701.583	687.791	98,03%	13.705	1,95%	87	0,0124%	Staphylococcus aureus (1)	NA
JE27d.1	1.345.879	1.315.258	97,72%	30.608	2,27%	13	0,0010%	Staphylococcus aureus (1)	Staphylococcus aureus (5)
JE27d.2	2.015.908	1.972.546	97,85%	43.273	2,15%	89	0,0044%	Staphylococcus aureus (1)	Staphylococcus aureus (3)
JE27e.1	1.057.894	1.024.653	96,86%	33.238	3,14%	3	0,0003%	NA	NA
JE27e.2	1.479.556	1.440.388	97,35%	39.166	2,65%	2	0,0001%	Staphylococcus aureus (1)	Staphylococcus aureus (1)
JE30a.1	4.835.565	4.637.115	95,90%	198.407	4,10%	43	0,0009%	Staphylococcus aureus (2)	Staphylococcus aureus (21)
JE30a.2	4.807.154	4.616.812	96,04%	190.297	3,96%	45	0,0009%	Staphylococcus aureus (3)	Staphylococcus aureus (22)
JE31a.1	4.795.032	4.338.927	90,49%	456.094	9,51%	11	0,0002%	Staphylococcus aureus (3)	Staphylococcus aureus (5)
JE31a.2	3.037.869	2.696.742	88,77%	341.113	11,23%	14	0,0005%	Staphylococcus aureus (4)	Staphylococcus aureus (8)
JE32a.1	5.666.184	5.053.074	89,18%	613.104	10,82%	6	0,0001%	Staphylococcus aureus (1)	Staphylococcus aureus (3)
JE32a.2	4.758.089	4.222.617	88,75%	535.467	11,25%	5	0,0001%	NA	NA
JE33a.1	9.890.185	9.356.618	94,61%	532.430	5,38%	1137	0,0115%	Staphylococcus aureus (191)	Staphylococcus aureus (1065)
JE33a.2	7.835.481	7.369.389	94,05%	465.179	5,94%	913	0,0117%	Staphylococcus aureus (135)	Staphylococcus aureus (852)
JE34a.1	1.686.222	1.522.471	90,29%	163.735	9,71%	16	0,0009%	Staphylococcus aureus (3)	Staphylococcus aureus (15)
JE34a.2	5.172.344	4.595.258	88,84%	577.022	11,16%	64	0,0012%	Staphylococcus aureus (6)	Staphylococcus aureus (60)
JE35a.1	5.797.113	5.064.443	87,36%	732.305	12,63%	365	0,0063%	Staphylococcus aureus (31)	Staphylococcus aureus (344)

Supplementary Methods

1. Blood Collection Tube Evaluation

To optimize the pre-analytical workflow, the performance of K3-EDTA tubes (Sarstedt) and Streck Cell-Free DNA BCT® tubes (Streck) for preserving mcfDNA was compared. Blood from healthy volunteers was collected in both tube types. For the first experiment, pooled plasma from each tube type was spiked with bacterial DNA after centrifugation. For the second experiment, whole blood was spiked with a bacterial mix and incubated for 3 hours at room temperature before plasma separation to simulate sample transport. Nuclease-free water was used as a no-template control (NTC) in each experiment to monitor for reagent contamination. DNA isolation was performed and total DNA yield was quantified via Qubit fluorometry.

Table S3: Comparison of total DNA yield from K3-EDTA and Streck BCT tubes.

The table shows the total amount of DNA recovered from plasma after spiking with known amounts of bacterial DNA. "DNA Input" refers to the mass of spiked bacterial DNA. "Total DNA Output" is the total DNA measured after isolation, which includes both the spiked bacterial DNA and the background host cfDNA.

Experiment	Tube Type	Sample Name	Spiking Condition	DNA Input (ng)	Total DNA Output (ng)
1	(NTC)	Water Control I	Nuclease-free water	0	ND
1	(Control)	Plasma I	Unspiked Plasma	0	11.2
1	Streck BCT	S 0.1 I	Spiked post-centrifugation (0.1 ng/µL)	200	108
1	Streck BCT	S 0.1 II	Spiked post-centrifugation (0.1 ng/µL)	200	130
1	Streck BCT	S 1 I	Spiked post-centrifugation (1.0 ng/µL)	2000	1130
1	Streck BCT	S 1 II	Spiked post-centrifugation (1.0 ng/µL)	2000	1020
1	(NTC)	Water Control II	Nuclease-free water	0	ND
1	K3-EDTA	E 0.1 I	Spiked post-centrifugation (0.1 ng/µL)	200	93
1	K3-EDTA	E 0.1 II	Spiked post-centrifugation (0.1 ng/µL)	200	90.4
1	K3-EDTA	E 1 I	Spiked post-centrifugation (1.0 ng/µL)	2000	994
1	K3-EDTA	E 1 II	Spiked post-centrifugation (1.0 ng/µL)	2000	1210
2	(NTC)	Water Control	Nuclease-free water	0	ND
2	(Control)	S0 I	Unspiked Streck BCT	0	ND
2	(Control)	E0 I	Unspiked K3-EDTA	0	ND
2	Streck BCT	Ss I	Spiked pre-incubation (1.0 ng/mL)	10	10.06

2	Streck BCT	Ss II	Spiked pre-incubation (1.0 ng/mL)	10	11.40
2	K3-EDTA	Es I	Spiked pre-incubation (1.0 ng/mL)	10	ND
2	K3-EDTA	Es II	Spiked pre-incubation (1.0 ng/mL)	10	ND

NTC: No-Template Control. **ND**: Not Detected. The DNA concentration was below the assay's limit of detection.
E: K3-EDTA tube **S**: Streck BCT

1. DNA Fragment Size Analysis

To characterize the size distribution of cell-free DNA (cfDNA) and validate the fragmentation of bacterial DNA used in spiking experiments, samples were analyzed using the Agilent TapeStation system with a High Sensitivity D1000 ScreenTape assay. The analysis included: (i) cfDNA isolated from the plasma of patients with infective endocarditis, (ii) cfDNA from healthy control donors, and (iii) genomic DNA from several bacterial species that was enzymatically fragmented. The purpose was to visualize the characteristic mononucleosomal peak of clinical cfDNA and to confirm that the fragmented bacterial DNA provided a suitable size range for use as a proxy in validation experiments.

Supplementary Fig. S1

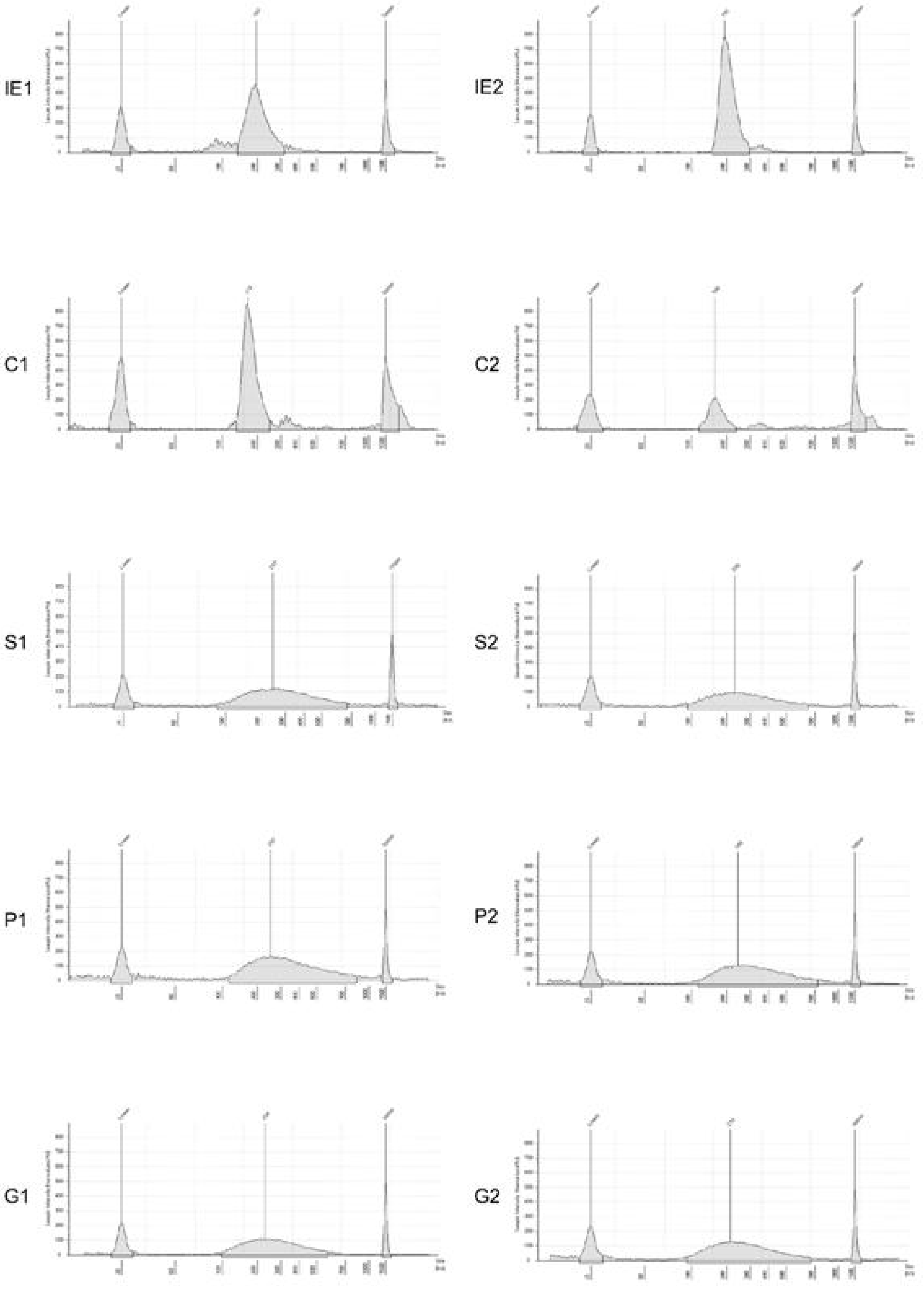

Fig. S1: Electropherograms of cfDNA and enzymatically fragmented bacterial DNA, measured using the Agilent TapeStation system. IE1 and IE2 represent cfDNA isolated from plasma of two patients with infective endocarditis; C1 and C2 show cfDNA from two healthy controls. The remaining samples (S1, S2, P1, P2, G1, G2) contain bacterial DNA enzymatically fragmented using NEB UltraShearTM Fragmentase to resemble cfDNA-like fragments. S1 and S2 correspond to *Staphylococcus aureus*, P1 and P2 to *Pseudomonas aeruginosa*, and G1 and G2 to *Geobacillus stearothermophilus*.

Supplementary Fig. S2

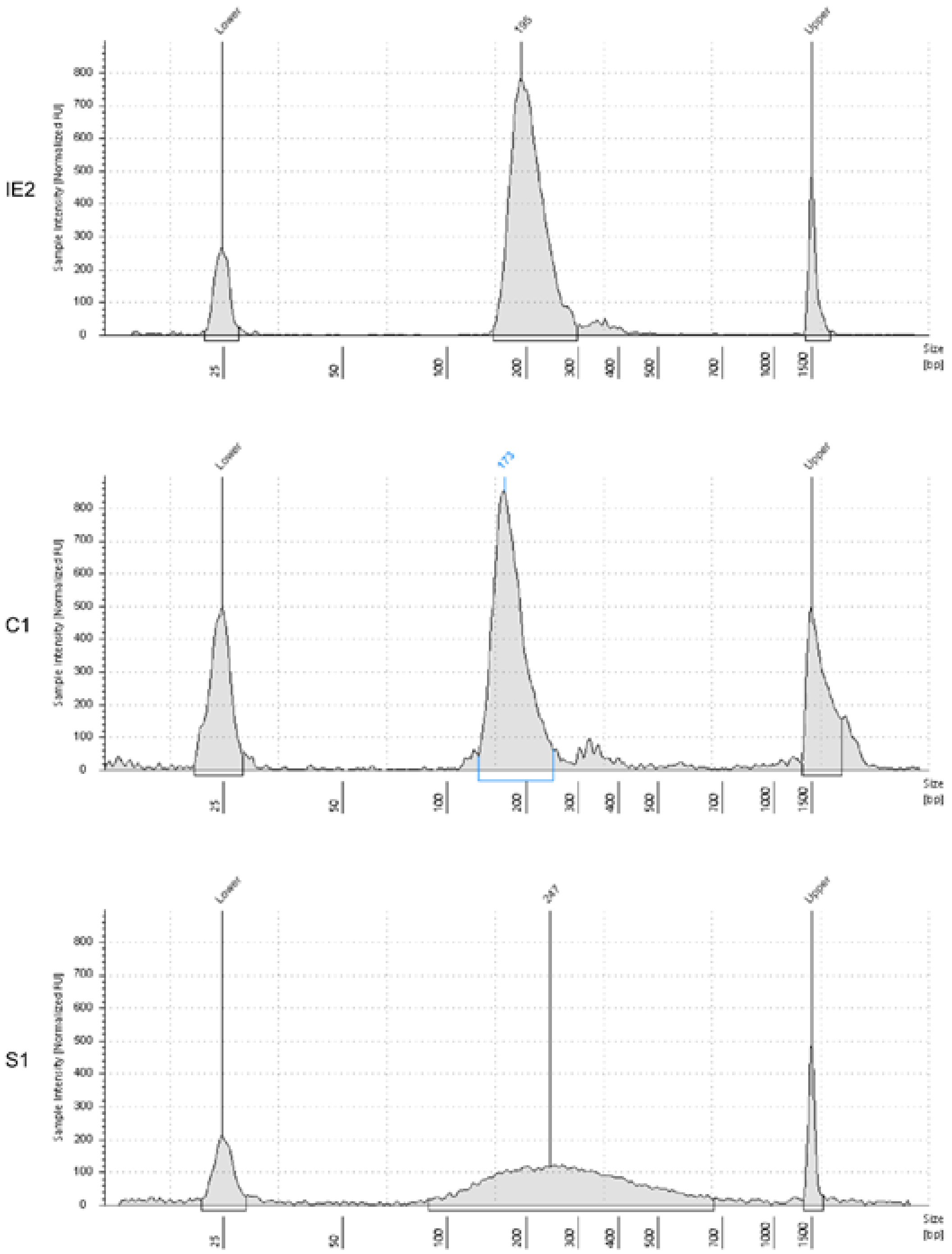

Fig. S2: Magnification of three representative samples. The figure shows magnified views of three key sample types to highlight their distinct size distribution profiles. Sample IE2 is cfDNA from a patient with infective endocarditis, showing a clean mononucleosomal peak. Sample C1 is cfDNA from a healthy control, illustrating

both the mononucleosomal peak and significant high molecular weight contamination. Sample S1 is enzymatically fragmented *Staphylococcus aureus* DNA, showing a broad distribution used as a proxy for mcfDNA.

Supplementary Fig. S3

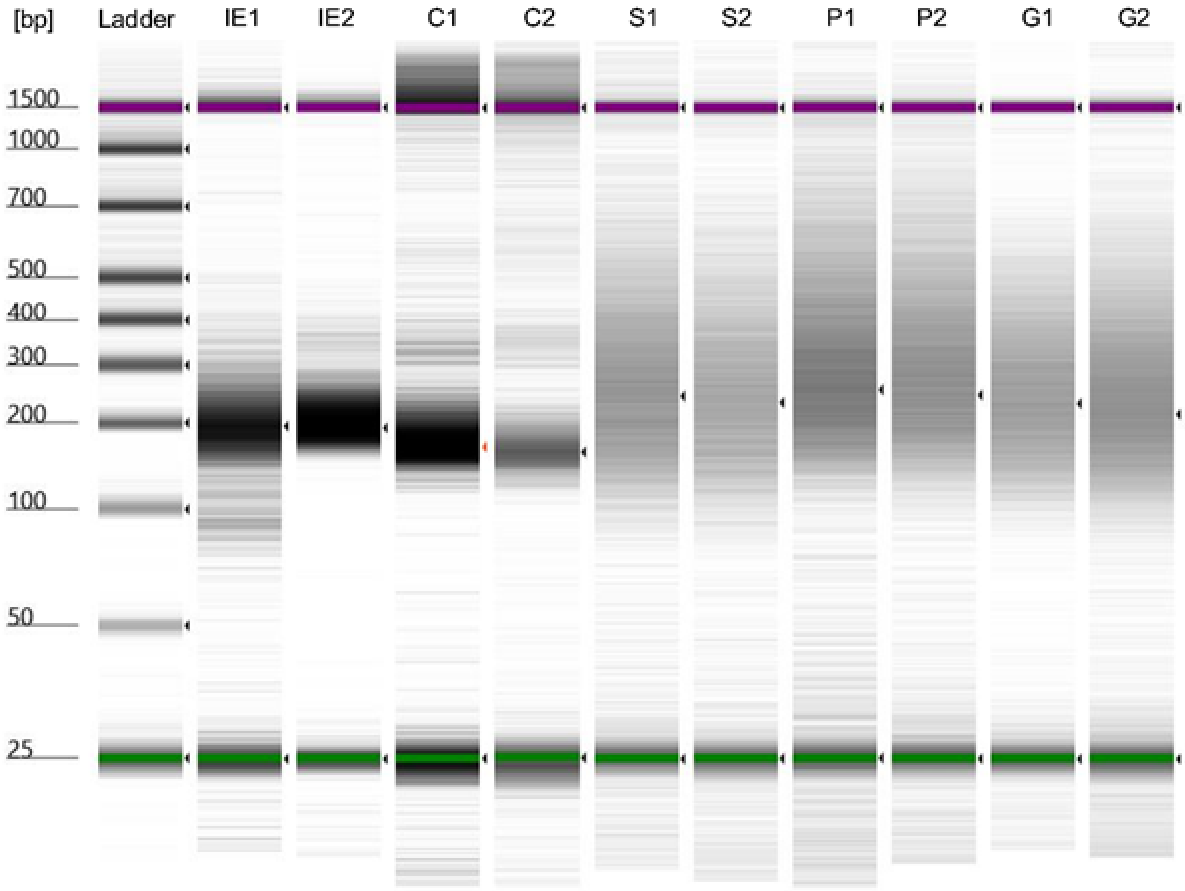

Fig. S3: Agilent TapeStation analysis of cfDNA and enzymatically fragmented bacterial DNA. This image provides a comparative overview of all ten DNA samples, visually contrasting the sharp cfDNA bands with the broad smears of the fragmented DNA. Lanes IE1 and IE2 show cfDNA isolated from plasma of two patients with infective endocarditis; lanes C1 and C2 show cfDNA from two healthy controls. The remaining lanes (S1, S2, P1, P2, G1, G2) contain the fragmented bacterial DNA. S1 and S2 correspond to *Staphylococcus aureus*, P1 and P2 to *Pseudomonas aeruginosa*, and G1 and G2 to *Geobacillus stearothermophilus*.

2. Statistical Limit of Detection (LOD) Calculation

To ensure maximum transparency, the empirical background noise and the resulting Limit of Detection (LOD) were calculated using a stringent 4-sigma (Z = 4) threshold. The baseline background noise was determined using mapping-confirmed reads from 9 independent negative control samples (6 nuclease-free water NTCs, 3 healthy volunteer plasma samples), yielding the following mapped-read counts: 0, 0, 2, 0, 0, 0, 0, 1, and 0.

The mean background noise (μ) across all controls was calculated as 0.33 reads. To account for the small sample size (n=9) and provide a conservative estimate of technical variance, Bessel's correction (n-1) was applied to calculate the sample standard deviation (s). The mathematical Limit of Detection was defined using the formula:

$$LOD = \mu + (4 \times s) = 0.33 + (4 \times 0.71) = 3.17 \text{ reads}$$

Because mapping-confirmed reads are discrete integers, only clinical samples with 4 or more reads ($Z \geq 4$) successfully clear this threshold and are considered statistically significant pathogen detections.

Supplementary Fig. S4

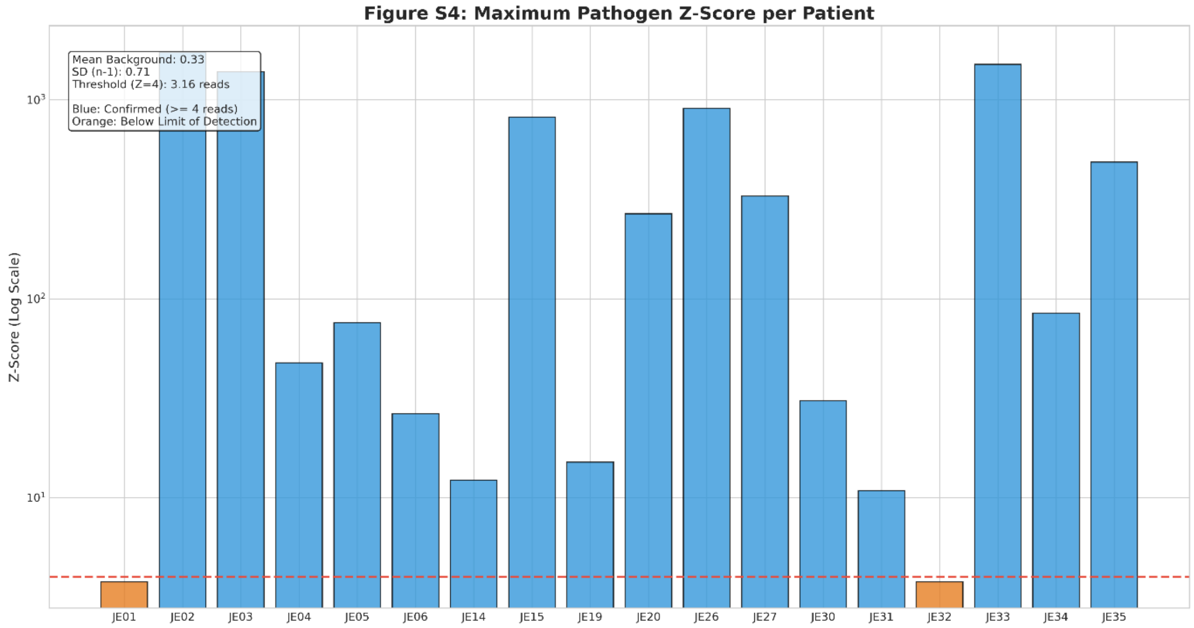

Fig. S4: Maximum statistical significance of pathogen detection per patient. The chart illustrates the calculated Z-scores for clinical samples plotted against the empirical background noise from 9 controls. To prioritize specificity and account for stochastic background, a stringent significance threshold of Z = 4 was implemented (red dashed line). Samples exceeding this threshold (blue bars) are considered statistically significant pathogen detections. Samples with 3 or fewer reads (e.g., JE01, JE32) remain below the Z = 4 limit (LOD = 3.17 reads) and are classified as non-significant relative to the empirical background noise (orange bars).
